# Editorial: Metabolic regulation under oxidative stress in cancer

**DOI:** 10.3389/fonc.2023.1286086

**Published:** 2023-09-12

**Authors:** Elavarasan Subramani, Abishai Dominic, Pratip K. Bhattacharya, Daniel E. Frigo, Ilya Bederman, Ali Vaziri-Gohar

**Affiliations:** ^1^ Department of Cancer Systems Imaging, The University of Texas MD Anderson Cancer Center, Houston, TX, United States; ^2^ Department of Genitourinary Medical Oncology, The University of Texas MD Anderson Cancer Center, Houston, TX, United States; ^3^ Center for Nuclear Receptors and Cell Signaling, University of Houston, Houston, TX, United States; ^4^ Department of Biology and Biochemistry, University of Houston, Houston, TX, United States; ^5^ Department of Genetics and Genome Sciences, Case Western Reserve University, Cleveland, OH, United States; ^6^ Department of Cancer Biology, Cardinal Bernardin Cancer Center, Stritch School of Medicine, Loyola University Chicago, Maywood, IL, United States

**Keywords:** oxidative stress, metabolism, cancer, therapy, biomarker

Oxidative stress has emerged as a key component of cancer metabolism that impacts multiple facets of tumor biology (1). Recent studies have shed light on the complex interplay of cellular redox and its impact on molecular mechanisms that govern metabolic reprogramming under oxidative stress (2, 3). It is well established that altered glucose metabolism exhibited by tumor cells leads to an enormous oxidative burden through various metabolic routes (4, 5). What is less known but remains a great interest to the field is whether elevated oxidative stress has a causal role in the development of aggressive and resistant tumor phenotypes (6). To that end, this Research Topic explores various metabolic routes that protect tumor cells against oxidative stress in diverse cancer models. It is anticipated that the development of advanced therapeutic approaches requires an in-depth understanding of the oxidative stress-mediated metabolic processes in cancer, including its effects on aspects of the tumor milieu such as immune cells-related functions.

The study by Koc et al. in this Research Topic identified the role of oxidative phosphorylation (OXPHOS) in ovarian cancer through mitochondrial proteomic analysis. They found that increased levels of mitochondrial- and nuclear-encoded OXPHOS subunits correlated with the increased rates of mitochondrial biogenesis in ovarian cancer cell lines. Furthermore, they identified that reduced OXPHOS subunits expression and mitochondrial translation significantly increased mitochondrial reactive oxygen species (ROS) production while decreasing superoxide dismutase 2 (SOD2). This study makes an interesting case for including mitochondrial biogenesis- and redox state-targeted therapies, which may have significant therapeutic impact when combined with current treatment strategies. To explore insights into the mitochondrial nucleic acids sensing signaling pathways, Gong et al. described a specific role of Y-box binding protein (YB1) with mitochondria-derived RNAs in breast cancer cell apoptosis and ROS production. As such, it is of great importance in exploring the inter-linkage of cytoplasmic and mitochondrial factors facilitating metabolic regulation for understanding redox metabolism in cancer. In an interesting study by Yuan et al. in this Research Topic, the research team has developed a prognostic risk model based on oxygen metabolism for colorectal cancer. Although this study was only a proof of concept for future studies of similar prognostic models, development of these correlative biomarkers will be useful in clinical cancer progression monitoring and can change the current management of colorectal cancer.

Glutathione peroxidases (GPX) protect cells from oxidative insults and they have been implicated in cancer progression and metastasis (7). In this Research Topic, Hu et al. showed that GPX3 was downregulated in several tumor types and that the expression levels correlate with patient outcomes. This finding suggests that GPX3 has a specific role in regulating redox states in cancer and could regulate the progression and metastasis of the disease. In another study by Canevarolo et al., increased glutathione (GSH) levels and other cellular oxidative stress mechanisms were found to be a key resistance mechanism to the antifolate methotrexate, a drug used for acute lymphoblastic leukemia (ALL). These findings point to another critical metabolic feature that mediates drug resistance in ALL.

Another rapidly emerging field in metabolism is ferroptosis, an iron-dependent cell death mechanism characterized mainly by substantial lipid peroxidation (8). Anti-ferroptotic mechanisms have been implicated in cancer progression and accordingly, inducers of ferroptosis are being actively pursued as a novel class of cancer treatments (9). In this special edition, Yuan et al. identified that STEAP3 gene is a key regulator of ferroptosis by evaluating patient data and molecular validation. Further, Han et al. developed a prognostic model for hepatocellular carcinoma and discovered a set of genes regulating ferroptosis mediated inflammatory responses. Similarly, Zhu et al. developed a 10-gene ferroptosis prognostic model in acute myeloid leukemia which might be of interest in future therapeutic targets. A more focused study by Yi et al. described the specific role of CXCL2 gene in ferroptosis related mechanisms and its negative association with clinical malignancy features. In search of new biomarkers and drug targets of hepatocellular carcinoma, Wen et al. revealed the mechanistic role of oncoprotein-induced transcript 3 protein (OIT3) regulating ferroptosis via arachidonic metabolism. Thus, OIT3 is suggested to be a potential diagnostic marker and therapeutic target of hepatocellular carcinoma.

In the recent years, there is a considerable increase in investigations on cuproptosis, a new form of copper-dependent regulated cell death and highly regulated by cellular metabolism (10). Zhang et al. found that 14 cuproptosis and copper metabolism-related genes significantly correlated with the immune microenvironment, suggesting its involvement in cancer progression. Their investigation suggested the potential use of cuproptosis and copper metabolism–related gene signatures as prognostic biomarkers of head and neck squamous cell carcinoma for better patient outcomes. Further, Wang et al. discussed the insights into copper metabolism and cuproptosis in cancer progression and its potential as targeted therapy. Although future studies are required to validate these initial findings of biomarker-based monitoring strategies, the studies covered in this Research Topic highlight the significance and potential impact in clinical settings.

Future investigation into the foundational mechanisms underlying metabolic signatures of tumor cells under oxidative stress will provide the knowledge needed to develop novel therapeutic strategies. In many cancer types, oxidative stress is highly elevated. Strategies leveraging this increased oxidative burden can lead to novel treatments such as those augmenting ferroptosis or cuproptosis. While much work has been done in this regard, an increased understanding of the molecular mechanisms underlying aberrant cellular metabolism and, in particular, oxidative stress in cancer progression and treatment resistance will enable the inception of new treatment strategies that leverage these tumor characteristics, ultimately yielding improved patient outcomes.

## Author contributions

ES: Writing – original draft, Writing – review & editing. AD: Writing – original draft, Writing – review & editing. PB: Writing – review & editing. DF: Writing – review & editing. IB: Writing – review & editing. AV-G: Writing – review & editing.
